# Plant Genomics—Advancing Our Understanding of Plants

**DOI:** 10.3390/ijms241411528

**Published:** 2023-07-16

**Authors:** Frank M. You

**Affiliations:** Ottawa Research and Development Centre, Agriculture and Agri-Food Canada, Ottawa, ON K1A 0C6, Canada; frank.you@agr.gc.ca

Plant genomics has made significant progress in recent years, enabling researchers to identify genes and genomic regions responsible for plant growth, development, and stress response. This Special Issue of Plant Genomics 2019 brings together 57 papers that provide insights into various aspects of plant genomics, including gene discovery, quantitative trait loci (QTL) identification, genomic prediction, genome editing, plant chloroplast genome sequencing and comparative analysis, microRNA analysis, and comparative genomics.

A comprehensive research approach combining bioinformatics and transcriptome analysis has been widely used in these studies to identify genes in response to various biotic and abiotic stress [1,2]. The approach includes (1) genome-wide identification of studied gene families from reference genomes and their annotations, bioinformatics analyses of identified genes such as chromosome distribution, gene structure, similarity and duplication, conserved domains and motif analysis, and phylogenetic analysis; and (2) expression profile analysis of different tissues in various development stages under different stress treatments using transcriptome data from Illumina RNA-Seq sequencing and/or real-time PCR analysis, and gene silencing in response to studied traits. Using this approach, in 22 papers, various gene families reported were studied to identify genes in response to abiotic stress, fruit ripening, seed development, seed yield, and pollen development in more than 12 species, such as tomato, wheat, *Eucalyptus grandis*, *Nicotiana tabacum*, grapevine, *Arabidopsis*, *Solanum lycopersicum*, cassava, *Brassica rapa*, *Gossypium hirsutum*, foxtail millet, and watermelon. The gene families include 2-oxoglutarate-dependent dioxygenase (2OGD), cytokinin oxidase/dehydrogenase (CKX), calcium-dependent protein kinase (CPK), karyopherin β, VQ, aquaporin, gibberellic acid stimulated Arabidopsis (GASA), YABBY transcription factor, B3-domain transcription factor, polygalacturonase (PG) and pectin methylesterase (PME), MADS-box transcription factor, WRKY transcription factor, teosinte-branched 1/cycloidea/proliferating (TCP) transcription factor, class III peroxidase (POD), glycoside hydrolase family 1 β-glucosidase, RNA editing factor, protein phosphatase (PP2C), LIM, brassinosteroid-signaling kinase (BSK), and chalcone synthase (CHS).

MicroRNAs (miRNAs) are small RNA molecules that play important regulatory roles in gene expression. Two papers have explored the role of miRNAs in different plant species. The first paper developed an artificial miRNA precursor system that allowed for efficient cloning and gene silencing in *Arabidopsis* and rice. This system could be a valuable tool for functional genomics studies in these crops [3]. The second paper identified and characterized miRNAs in the developing seed of linseed flax, an important oilseed crop [4]. The results showed that miRNAs play important roles in seed development and could be targeted for crop improvement. Overall, these studies contribute to our understanding of the regulatory roles of miRNAs in plant growth and development and suggest potential applications for crop improvement.

GWAS has been widely used to identify QTLs or quantitative trait nucleotides (QTNs) associated with important traits in plants. One of the exciting papers in this issue was on the haplotype networking of GWAS hits for citrulline variation associated with the domestication of watermelon [5]. This paper identified the genomic regions that control the synthesis of citrulline, a non-proteinogenic amino acid that plays a vital role in the watermelon’s fruit quality and nutritional value. The authors used a combination of genome-wide association mapping and haplotype-based network analysis to uncover the genetic basis of citrulline variation in wild and domesticated watermelons. This study not only provided insights into the domestication history of watermelons but also revealed the potential for using genomic tools to enhance the nutritional quality of crops.

One of the major themes of this Special Issue is the application of genomic tools for crop improvement. The papers on wheat, flax, rice, and tomato, for instance, demonstrate the effectiveness of genomic prediction and marker-assisted selection for enhancing yield, salt tolerance, and fruit ripening. Two papers in this issue focused on the genomic prediction of agronomic traits and yield parameters through molecular markers and QTLs in wheat [6] and flax [7]. For example, a paper on genomic prediction for grain yield and yield-related traits in Chinese winter wheat reported a high accuracy of genomic prediction for several agronomic traits, including grain yield, plant height, and spike length [6]. In addition, the paper on salt tolerance improvement in rice shows how SNP marker-assisted selection can be combined with speed-breeding to rapidly develop new, more resilient varieties of this important staple crop [8].

Genome editing technologies such as CRISPR/Cas9 have revolutionized the field of plant genomics and provided researchers with powerful tools to precisely modify plant genomes. In this Special Issue, one review paper [9] provides a comprehensive overview of the current status of CRISPR/Cas9-mediated genome editing in plants, including the development of efficient delivery systems, targeted mutagenesis, and gene replacement. The authors also discuss the challenges and future prospects of CRISPR/Cas9-mediated genome editing in plants.

The papers in this issue also highlight the importance of comparative genomics in understanding the evolution of plant species. By comparing the genomes of different plant species, researchers can identify conserved gene families and regulatory elements that have been conserved across long periods of evolutionary time. This approach has led to the discovery of new gene families that are involved in plant development and adaptation to environmental stressors. For example, the complete chloroplast genomes of *Punica granatum* and seven *Aristolochia* medicinal species were compared to gain insights into the evolution of these species [10,11]. The complete chloroplast genome can substantially increase species discriminatory power and resolve phylogenetic relationships in oaks (*Quercus* L., *Fagaceae*) [12].

In summary, the 57 papers in this Special Issue of Plant Genomics 2009 cover a wide range of topics in plant genomics, highlighting the significant progress that has been made in recent years in understanding the genetics and genomics of plant growth, development, and stress response. These studies provide valuable insights into the potential applications of genomic tools and technologies for crop improvement and sustainable agriculture, as well as fundamental questions about the evolution and function of plant genes and genomes.
